# Invasive fungal infections in renal transplant patients: a single center study

**DOI:** 10.1080/0886022X.2016.1268537

**Published:** 2017-01-13

**Authors:** Minaxi H. Patel, Rashmi D. Patel, Aruna V. Vanikar, Kamal V. Kanodia, Kamlesh S. Suthar, Lovelesh K. Nigam, Himanshu V. Patel, Ansy H. Patel, Vivek B. Kute, Hargovind L. Trivedi

**Affiliations:** a Department of Pathology, Laboratory Medicine, Transfusion Services and Immunohematology, IKDRC-ITS, Ahmedabad, India;; b Department of Nephrology and Clinical Transplantation, Institute of Kidney Diseases and Research Center, Dr H.L. Trivedi Institute of Transplantation Sciences (IKDRC-ITS), Ahmedabad, India

**Keywords:** Renal transplant, Invasive fungal infection, *Candida albicans*, Aspergillus, Mucor

## Abstract

**Background:** Timely diagnosis of invasive fungal infections (IFI) in renal transplant (RT) patients on immunosuppression is often difficult, jeopardizing their life and graft. We reported IFI and their causative fungal agents in post-RT patients.

**Materials and methods:** This was a retrospective 6-year clinical study carried out from 2010 to 2015 on 1900 RT patients. Clinical data included patient-donor demographics, time to onset of infection, risk factors and graft function in terms of serum creatinine (SCr). To identify IFI, we examined bronchoalveolar lavage (BAL), blood, tissue, and wound swab samples by conventional mycological methods.

**Results:** IFI were diagnosed in 30 (1.56%) patients on triple immunosuppression, mainly males (*n* = 25) with mean age of 36.57 ± 11.9 years at 13.12 ± 18.35 months post-RT. *Aspergillus* species was identified in 11 BAL, one tissue, and one wound specimen each, 30.76% of these were fatal and 15.38% caused graft loss; *Candida albicans* was in nine BAL, four blood, two wound swab, and one tissue specimens, 25% of these were fatal and 25% had graft loss and one mucor in BAL which was fatal. Seven patients were diabetic, 10 had superadded cytomegalovirus infection, and 15 were anti-rejected.

**Conclusion:** IFI are associated with increased morbidity and mortality in RT patients. Triple immunosuppression, broad spectrum antibiotics for ≥ two weeks, diabetes and superadded infection are added risks for these patients. Prevention, early diagnosis, and appropriate management are necessary to improve their prognosis.

## Introduction

A few decades ago, renal transplantation (RT) was less preferable as compared to dialysis for patients with end stage renal diseases (ESRD) because of technical complications, rejections and complications like systemic infections secondary to immunosuppression. Now with advancement in the field of transplantation biology and research, transplantation has become the most accepted and effective means of rehabilitating these patients. This does not imply that all the problems have been solved; however, results in terms of graft and patient survival have improved remarkably. Infections are the major source of morbidity and mortality in RT recipients (RTR) due to long term, graft-preserving immunosuppressive therapy predisposing them to infections, including fungal infections. Fungal infections account for 5% of all infections in RTR. The incidence varies according to geographical area, because of environmental exposure and the effects of immunosuppressive regimens.^1^ Systemic mycosis is a significant problem in transplant patients worldwide and remains the major cause of death in these individuals.^2^ In an Indian study, 6.1% of RTR were affected by systemic fungal infections and resulted in 63% mortality rate.^3^ Transplant immunosuppressive therapy primarily targets T-cell-mediated graft rejection. Calcineurin inhibitors (CNI), which include cyclosporine and tacrolimus, impair calcineurin-induced up-regulation of IL-2 expression, resulting in increased susceptibility to invasive fungal infections (IFI). This immunosuppressive state allows infectious complications leading to a high mortality rate. Currently, overall mortality due to IFI in solid organ transplant (SOT) recipients ranges between 25 and 80%. Most fungal infections occur in the first 6 months after transplantation due to optimum dose of various immunosuppressive medications. *Candida* and *Cryptococcus* species are the most frequently isolated yeasts, while most frequent filamentous fungi (molds) isolated are *Aspergillus* species. The symptoms of systemic fungal infections are nonspecific. Early detection and appropriate timely management of fungal infections plays decisive role in improving the survival and reducing mortality.^4^ Various studies have reported variable presentations and outcomes of fungal infections after RT.^5–17^ This study was carried out in a single center to find out the prevalence and natural history of IFI in RTR, diagnostic procedures, treatment modalities adopted, and presents their final outcome.

## Material and methods

This was a single-center, retrospective observational study of 1900 RTR who underwent RT between 2010 and 2015. Medical records were studied. Diagnosis was based on clinical presentation, radiological findings and unresponsiveness of infections to conventional antibiotic therapy followed by microbiological findings. Response to antifungal treatment was recorded. Patient-donor demographics evaluated included age, gender, date of transplantation, type of donor (living or deceased), HLA-matching, time to onset of infection, and clinical presentation in the form of fever, respiratory/gastrointestinal/neurological/urogenital involvement, immunosuppressive therapy, and graft function in terms of serum creatinine (SCr). Specimens subjected to culture and sensitivity were sputum, bronchoalveolar lavage (BAL), wound infections, tissue, and blood. Culture sensitivity was carried out in Bactec 9050, (BD). Samples were subjected to culture medium for a maximum of seven days and those which did not show any growth were reported as negative for fungal isolates.

## Identification of fungus

Aspergillosis was diagnosed by identification of hyphomycete showing distinctive conidial heads with flask-shaped phialides arranged in whorls on a vesicle. These were isolated from specimens of BAL fluid and by galactomannan antigen testing. *Candida* species were diagnosed by special stains showing budding yeasts and pseudohyphae or hyphae. *Candida* species were identified by positive blood culture (*n* = 4) or BAL culture (*n* = 9). The beta-D-glucan assay was useful adjunct test if required. Mucor were identified by examination of sputum and BAL specimens (*n* = 1), which showed characteristic broad non-septate hyphae, which is often the first indicator of mucormycosis.

Chest radiographs and CT scans demonstrated a reversed halo sign (a focal area of ground glass attenuation surrounded by a ring of consolidation) characteristic of angioinvasive fungal infections.

### Treatment

Upon diagnosis, all patients were treated with intravenous liposomal amphotericin-B. The initial dose was 1 mg/kg, which was gradually increased to 3–5 mg/kg with close observation for side effects for a total duration of 3–4 weeks. Simultaneously, the dose of the immunosuppressive drugs was reduced in all cases. Mycofenolate was discontinued and dose of CNI was reduced by half.

## Result

### Demographics

IFI were diagnosed in 30 (1.56%) patients with mean age of 36.57 ± 11.9 years (range 15–58); 25 were males and five were females. The mean donor age was 48.17 ± 13.52 years (range: 27–70); 25 donors were females and five were males. Majority of donors (*n* = 25) were living related, while five were deceased donors. Mean HLA-matching (A, B, DR, DQ) was 1.96 ± 1.30 (range: 0–4/6).

### Presentation and risk factors for IFI

Mean time from RT to infection was 13.12 ± 18.35 months and mean time from fungal infection to outcome (death/graft loss/recovery) was 57 ± 60 days (5–228 days). Prolonged fever of ≥100 °F for seven days not responding to antibiotics was the most common presentation followed by accompanying pulmonary manifestations. All patients were on triple immunosuppression therapy with CNI, mycophenolate, and prednisolone at time of IFI and received broad spectrum antibiotics for ≥ two weeks before IFI. Cumulative dose of rabbit-antithymoglobulin (r-ATG, Thymoglobuline^®^, Genzyme) was ≥3 mg/kgBW in all patients and ≥4 mg/kgBW in all patients who succumbed to infection (*n* = 6). Other risk factors for IFI were diabetes in seven (23.3%) patients, superadded cytomegalovirus infection in 10 (33.3%) patients, and anti-rejection therapy in 15 (50%) patients.

### Fungal species and site of IFI

Fungal species isolated were *Candida albicans* [53.30%(*n* = 16)], *Aspergillus fumigates* [36.70%(*n* = 11)], *Aspergillus niger* [6.70%(*n* = 2)] and Mucor [3.30%(*n* = 1)].

Invasive aspergillosis was diagnosed in 13 patients. *Apergillus* species was identified in 11 BAL, one renal graft site, and one wound infection specimen. Out of 13 patients with aspergillus infection, four (30.76%) died of IFI, and two (15.38%) had graft loss. *Candida* species was identified in nine BAL, four blood, two wound swabs, and one graft specimen. Four (25%) patients succumbed to infection and four (25%) had graft loss. Mucor was isolated in one patient from BAL who had pneumonia. The patient was treated with amphotericin B, however, the patient died due to septic shock.

### Treatment and outcome

Mean SCr at the time of diagnosis of IFI was 1.6 ± 0.9 mg/dL and at last follow up was 2.06 ± 1.34 (0.72–5.68) mg/dl. Upon diagnosis, all patients were treated with intravenous liposomal amphotericin-B. The initial dose was 1 mg/kg, which was gradually increased to 3–5 mg/kg with close observation for side effects for a total duration of 3–4 weeks. Simultaneously, the dose of the immunosuppressive drugs was reduced in all cases. Mycofenolate was discontinued and dose of CNI was reduced by half.

Improvement was observed in 21 (70%) patients. CNI were restarted in low dose in 15 patients after clinical and laboratory control of IFI and 15 patients were left on low-dose steroid monotherapy.

## Discussion

The incidence of IFI following SOT ranges from 5 to 42% and varies with the organ being transplanted. *Candida* and *Aspergillus* species are the leading causative agents, with the median time to onset following transplantation depending on the type of transplant.^18^ These infections are associated with high overall mortality.^19^ The antifungal prophylaxis regimen currently in use varies among institutions. The overall efficacy of antifungal prophylaxis in SOT recipients was evaluated in a meta-analysis of 14 randomized trials with 1497 participants.^20^ They reported that antifungal prophylaxis did not reduce mortality. In RTR, neither ketoconazole nor clotrimoxazole significantly reduced IFI. Patients who should be considered for antifungal prophylaxis include those subjected to anti-rejection therapy, known fungal colonization pre-transplantation, prior (broad-spectrum) antimicrobial use, CMV infection, large blood transfusion requirements, prolonged intensive care unit stay, and renal and hepatic dysfunction. Antifungal prophylaxis regimens vary among institutions.^21^ Gaps in antifungal coverage should be noted for the echinocandins and azoles. Echinocandins have no activity against Mucorales/moulds or *Cryptococcus neoformans*; fluconazole, itraconazole, and voriconazole have no activity against Mucorales/moulds.

Fluconazole appears to be safe and has not been associated with nephrotoxicity following RT; it can be used as prophylaxis (100 mg once a day for 3 months) against susceptible *Candida* species and reduces invasive infections in such patients.^20^ Fluconazole does not have activity against filamentous fungi. In addition, some *Candida* species have relative resistance (high minimum inhibitory concentrations to the drug). Drug interactions with CNI are variable, however, they are known to increase the bioavailability of CNI in most patients. Similarly, serum CNI levels fall when prophylaxis is discontinued; dose readjustment is essential to prevent graft rejection (Table 1).^5–10^

**Table 1.** Systemic fungal infections: comparative data.^5–10^

	Gallis et al.^5^	Nampoory et al.^6^	John et al.^7^	Jaykumar et al.^8^	Gupta et al.^9^	Chugh et al.^10^^,^^12^
Fungal infection (%)	13	3.7	5.6	19	9.8	6.1
Candidiasis (%)	2.3	1.6	1.4	13.8	2.8	37
Cryptococcosis (%)	5.8	0.5	2.4	0.8	1.9	42
Aspergillosis (%)	1.2	0.9	1	3	2.3	5.5
Mucormycosis (%)	1.2	0.4	1.1	1.5	2	11
Others (%)	0.6	–	0.9	–	0.5	5.5

The mortality rate in the present series was low (30%), which could be due to early diagnosis and treatment.^17^ Amongst 310 live related RTR, systemic fungal infections were observed in 19 patients (6.1%). These included cryptococcosis [42%], candidiasis [37%], mucormycosis [11%], aspergillosis [5.5%], and a mixed cryptococcal and Aspergillus infection [5.5%]. Infections occurred within 12 months of RT in seven patients and after 13–37 months in the remaining patients.^10^ Godara et al.^11^ reported 16 patients of mucormycosis out of 1330 RT patients, between 2005 and 2009. The site of mucormycosis was rhinocerebral [56.25%], pulmonary [31.25%], disseminated mucormycosis and graft infection [6.25%] and six patients died. One patient died despite graft nephrectomy for graft site mucormycosis. Recipients of solid organ transplants have 24–40% incidence of opportunistic fungal infections with a very high mortality of 70–100%.^10^ This is related to the environmental exposure and net state of immunosuppression.^12^

Renal transplant recipient, who developed gastric mucormycosis along with tissue invasive CMV disease, within 4 weeks of renal transplant and was diagnosed on the basis of upper GI endoscopy and gastric biopsy, has been reported. The patient succumbed to the infection in spite of gastrectomy, antifungal, and antiviral therapy.^13^

Data from 1476 primary renal-transplant recipients was prospectively recorded from 1986 to 2000 at a single center. A total of 110 episodes of systemic mycoses occurred in 98 patients. The fungal genera Aspergillus, Cryptococcus, and Candida constituted 61% of pathogens, 45% localizing to the lungs. The probability of survival with systemic mycoses was 73, 60, 39, and 25% and was 92, 87, 80, and 75% without systemic mycoses at 1, 2, 5, and 10 years, respectively.^16^

### Mycoses in RTR in the Indian scenario

In a recently published study, prevalence of systemic mycoses was reported as 6.6% from Southern India similar to that in North India.^14–17^ Reports from western countries reveal a varying prevalence from 1.4 to 9.4%. This difference with the west is due to less intense immunosuppression resulting in lower systemic mycoses in western countries and the presence of poor hygienic and diagnostic facilities in developing countries. The risk factors for mycoses include CMV disease, chronic liver disease, hyperglycemia, and tuberculosis, and post-transplant period with cyclosporine.^14–16^ The overall probability of survival was poor; however, survival has recently improved.^16^ The major pathogens implicated here are *Aspergillus* (recently on upsurge), *Cryptococcus*, and *Candida* with 45% localizing to lungs.^16^ Gupta et al.^9^ reported 9.8% post-transplant patients who had systemic mycoses with candidiasis (2.8%), aspergillosis (2.3%), mucormycosis (2%), and cryptococcosis (1.9%). He also reported a recent rise in angio-invasive infections like aspergillosis and mucormycosis, which are associated with high mortality.

Our results were in conformity with the reports from other centers in the country and abroad.^5–8^ Unusual fungal infections and their manifestation in the renal transplant population has been reported.^22^ Two of them have etiological agents (Aspergillus), which are common among immunosuppressed patients, but with an atypical clinical presentation, while one of them is a subcutaneous infection caused by a less frequent dematiaceous fungus, Aureobasidiumpullulans.

Liposomal Amphotericin B is definitely less nephrotoxic than normal Amphotericin B. It is broad spectrum antifungal drug. Overall, the probability of survival with systemic mycoses was poor; however, survival has recently improved with prevention, early diagnosis and appropriate management.^16^

The strength of this study is that it may help to identify early post-renal transplant patients at high-risk of death from IFI. Also, to our knowledge, this is the largest single center study reported from India on IFI in renal transplant patients. The main limitation of this study is that this was a retrospective, single-center, observational study and small sample size. Multi-center, prospective, controlled clinical trials in a larger cohort are needed to further substantiate our observations.

## Conclusion

IFI is a rare complication following RT. IFI is associated with increased morbidity and mortality in RT patients. Triple immunosuppression, broad spectrum antibiotics for ≥ two weeks, anti-rejection therapy, diabetes, and superadded infection are added risks for IFI. Prevention, early diagnosis, and appropriate management are necessary to improve their prognosis.
